# 859. The Role of Procalcitonin in Antimicrobial De-escalation and Stewardship Program in Febrile Neutropenic Cancer Patients

**DOI:** 10.1093/ofid/ofad500.904

**Published:** 2023-11-27

**Authors:** Hiba Dagher, Anne-Marie Chaftari, Ray Y Hachem, Ying Jiang, Ann Philip, Patricia Mulanovich, Andrea Haddad, Peter Lamie, Rita Wilson Dib, Teny John, Natalie J Dailey Garnes, Patrick Chaftari, Issam I Raad

**Affiliations:** UT MD Anderson Cancer Center, Houston, Texas; MD Anderson UT, Houston, Texas; MD Anderson UT, Houston, Texas; The University of Texas MD Anderson Cancer Center, Houston, Texas; MD Anderson Cancer Center, Houston, Texas; MD Anderson Cancer Center, Houston, Texas; UT MD Anderson Cancer Center, Houston, Texas; UT MD Anderson Cancer Center, Houston, Texas; MD Anderson Cancer Center, Houston, Texas; MD Anderson Cancer Center, Houston, Texas; University of Texas MD Anderson Cancer Center, Houston, TX; UT MDAnderson Cancer Center, Houston, TX; MD Anderson UT, Houston, Texas

## Abstract

**Background:**

Serial procalcitonin (PCT) measurement has been adopted as an adjunct to clinical judgement to guide antibiotic therapy, prevent antibiotics overuse and endorse antimicrobial stewardship programs. PCT peaks at 24 to 48 hours, and declines with infection resolution. In this prospective study we explored the role of PCT used as a biomarker in adjunct to the clinical judgment to guide antibiotic course.

**Methods:**

We prospectively enrolled cancer patients with febrile neutropenia (FN) to receive empiric intravenous broad-spectrum antibiotics (IV-BSA) for at least 48 hours. PCT was measured at baseline and 48-72 hours after initiation of study drug. A PCT drop at 48-72 hours after study drug initiation was defined as a drop by ≥30 % from baseline (start of therapy) or a level of < 0.25 ng/ml. De-escalation was defined as a switch of IV-BSA to oral or simplified once a day IV therapy.

**Results:**

A total of 80 patients were enrolled between October 2021 and April 2023, of whom 74 had serial PCT measurements. Median age was 59 years, 57% were male, and 66% had hematological malignancies. A PCT drop at 48-72 hours was observed in 68% of patients who de-escalated to oral or IV therapy. In this PCT guided study, the median time to de-escalation was 62 hours (< 3 days). Furthermore, 64% of these patients de-escalated within 3 days after initiation of therapy, 79% de-escalated by day 4, 85% by day 5 and 92% by day 7. Patients with PCT drop had a shorter median duration of antibiotic compared to those without PCT drop (54.8 hours vs 106.4 hours; p=0.003). Furthermore, the antibiotic therapy de-escalation occurred sooner in patients with PCT drop (75% occurred prior or at 72 hours in the PCT drop group vs 41% in the non-PCT drop group; p=0.017). Patients who had a documented bacteremia had a significantly higher median PCT level at baseline than those without bacteremia (3.92 ng/ml vs 0.37 ng/ml; p=0.001).

**Conclusion:**

In this PCT guided study, the median time to de-escalation of IV-BSA was < 3 days and most of the cancer patients with FN who de-escalated had a PCT drop within 3 days of initiating therapy. Patients with a PCT drop had an earlier de-escalation and a shorter course of antibiotic therapy.

**Disclosures:**

**Natalie J Dailey Garnes, MD, MPH**, AlloVir: Grant/Research Support **Issam I. Raad, Distinguished Professor**, Novel Anti-Infective Technologies, LLC: Technology License

